# Can Gender-Fair Language Reduce Gender Stereotyping and Discrimination?

**DOI:** 10.3389/fpsyg.2016.00025

**Published:** 2016-02-02

**Authors:** Sabine Sczesny, Magda Formanowicz, Franziska Moser

**Affiliations:** Department of Psychology, University of BernBern, Switzerland

**Keywords:** gender stereotypes, gender-fair language, social discrimination, gender equality, social change

## Abstract

Gender-fair language (GFL) aims at reducing gender stereotyping and discrimination. Two principle strategies have been employed to make languages gender-fair and to treat women and men symmetrically: neutralization and feminization. Neutralization is achieved, for example, by replacing male-masculine forms (*policeman*) with gender-unmarked forms (*police officer*), whereas feminization relies on the use of feminine forms to make female referents visible (i.e., *the applicant… he or she* instead of *the applicant… he*). By integrating research on (1) language structures, (2) language policies, and (3) individual language behavior, we provide a critical review of how GFL contributes to the reduction of gender stereotyping and discrimination. Our review provides a basis for future research and for scientifically based policy-making.

Linguistic gender asymmetries are ubiquitous, as documented in the contributions in Hellinger and Bußmann (2001 2002, 2003), which analyze 30 languages (e.g., Arabic, Chinese, English, Finnish, Hindi, Turkish, Swahili) from various language families. An almost universal and fundamental asymmetry lies in the use of *masculine generics*. In English, for example, generic *he* can be used when gender is irrelevant (e.g., *the user… he*) and in German, masculine role nouns serve as labels for mixed gender groups (e.g., *einige Lehrer*, masc.pl ‘several teachers’ for a group of male and female teachers). Thus, masculine forms not only designate men but also mixed-gender groups or referents whose gender is unknown or unspecified (see Stahlberg et al., 2007). Feminine forms, on the other hand, do not function generically but refer to women only (Hellinger and Bußmann, 2001).

That masculine forms are used to represent all human beings is in accord with the traditional gender hierarchy, which grants men more power and higher social status than women (Ridgeway and Correll, 2004). A large-scale content analysis of 800,000 Reuters news messages (published in English between 1996 and 1997) found that the pronoun *he* was more frequent than *she* in the news and also appeared in more positive contexts (Gustafsson Sendén et al., 2014). The interrelation of language and the gender hierarchy has also been documented in a study which analyzed the ratio of male to female pronouns (e.g., *he/she*, *his/hers*) in written texts (full texts of about 1.2 million U.S. books, years 1900–2008; from the Google Books database; Twenge et al., 2012). This ratio was found to reflect the status of women in the United States during the 20th century. When women’s status was high (as indicated by educational attainment, labor force participation, etc.), the proportion of female pronouns was higher; when women’s status was low, female pronouns were less frequent.

Gender-fair language (GFL)^1^ was introduced as a response to this structural asymmetry and as part of a broader attempt to reduce stereotyping and discrimination in language (see Fairclough, 2003; Maass et al., 2013, for the political correctness debate). GFL aims to abolish asymmetries in referring to and addressing women and men, for example, by replacing masculine forms (*policeman*) with gender-unmarked forms (*police officer*), or by using both masculine and feminine forms (i.e., *the applicant… he or she* instead of *the applicant… he*).

In this paper, we review theoretical and empirical work on the role of GFL in sustaining or reducing gender stereotyping and social discrimination, as a follow-up on a comprehensive research program (the *Marie Curie Initial Training Network - Language, Cognition, and Gender, ITN LCG*, http://www.itn-lcg.psy.unibe.ch/content/index_eng.html). In this framework, we survey research on (1) language structures, (2) language policies, and (3) individual language behavior in order to draw conclusions on the effectiveness of GFL and to identify boundary conditions and obstacles for its implementation. Our aim is to critically discuss and integrate research findings to answer the question of whether and under what circumstances GFL contributes to the reduction of gender stereotyping and discrimination. Hopefully, this review will provide a useful basis for future research and for scientifically based policy-making.

## Language Structures

Although gender asymmetries exist in most, if not all, languages, they may be more or less conspicuous, depending on the structure of the language. Three types of languages can be distinguished: grammatical gender languages, natural gender languages, and genderless languages (see Stahlberg et al., 2007). **Table 1** gives an overview of this typology, describing the main characteristics of the different types with regard to gender and gender asymmetries as well as preferred strategies of linguistic gender-fairness. German, French, and Czech, for example, are *grammatical gender languages*. In these languages, every noun has a grammatical gender and the gender of personal nouns tends to express the gender of the referent. In *natural gender languages* (English or Swedish)^2^ personal nouns tend to be gender-neutral (e.g., *neighbor*) and referential gender is expressed pronominally (e.g., *he/she*). In *genderless languages* such as Finnish or Turkish neither personal nouns nor pronouns signal gender. Here, gender is only expressed through attributes such as ‘male/female [teacher]’ or in lexical gender words such as ‘woman’ or ‘father.’ Consequently, gender and linguistic gender asymmetries are much more visible in grammatical gender languages than in natural gender languages or genderless languages (Hellinger and Bußmann, 2001).

**Table 1:** Overview of language types regarding expression of gender and gender asymmetries.

Language type	Characteristics	Visibility of gender and gender asymmetries	Preferred strategies for gender-fair language
(1) Genderless (e.g., Finnish, Turkish)	• Neither personal nouns nor pronouns differentiated for gender (e.g., Turkish *öǧrenci* ‘student,’ *o* ‘she/he’) • Gender expressed only lexically via attributes (e.g., ‘male/female [student]’) or lexical gender nouns (e.g., ‘woman,’ ‘father’)	• Referential gender often not explicit • (Lexical) gender asymmetries exist, but are less frequent than in (2) and (3) Examples: Turkish *adam* ‘man’ and ‘human being’ Finnish job titles ending in -*mies* ‘-man,’ *lakimies* ‘lawyer,’ *lehtimies* ‘journalist’	GFL policies generally deemed unnecessary
(2) Natural gender (e.g., English, Swedish)	• Most personal nouns gender-neutral (e.g., *neighbor*, *student*) • Personal pronouns differentiated for gender (e.g., Swedish *hon/han* ‘she/he’)	• Referential gender more often explicit than in (1), but less often than in (3) • Lexical and pronominal asymmetries exist, but are less frequent than in (3) Examples: English *chairman*, *the typical student … he*	Neutralization
(3) Grammatical gender (e.g., French, German)	• Every noun has grammatical gender • Gender of personal nouns tends to match gender of referent (e.g., German *Student*_masc_/*Studentin*_fem_ ‘male/female student’) • Personal pronouns differentiated for gender (e.g., German *sie/er* ‘she/he’) • Pronouns and other grammatically dependent words signal gender of personal noun (e.g., *der*_masc_ *Student*_masc_ ‘the (male) student’ *eine*_fem_ *kluge*_fem_ *Studentin*_fem_ ‘a clever (female) student’)	• Referential gender often explicit • All kinds of asymmetries exist and are more frequent than in (1) and (2) Examples: French *homme* ‘man’ and ‘human being’ German *der*_masc_ *typische Student*_masc_ … *er* ‘the typical student (masc) … he’ German *alle Wähler*_masc_ ‘all voters’	Feminization + Neutralization

The way gender is encoded in a language may be associated with societal gender equality (Stahlberg et al., 2007). This assumption was tested empirically for 111 countries with different language systems, controlling for geographic, religious, political, and developmental differences (Prewitt-Freilino et al., 2012). In this research, the *Global Gender Gap Index* of the *World Economic Forum* was used to determine gender equality (GGI; Hausmann et al., 2009). Countries with grammatical gender languages were found to reach lower levels of social gender equality than countries with natural gender languages or genderless languages. This suggests that a higher visibility of gender asymmetries is accompanied by societal gender inequalities. A survey on sexist attitudes yielded additional evidence for this relationship (Wasserman and Weseley, 2009): respondents (native speakers of English as well as bilinguals) exhibited more sexist attitudes when the survey was conducted in a grammatical gender language (Spanish or French) than in a natural gender language (English). These findings document that, from the perspective of gender-fairness or gender equality, grammatical gender languages present a particularly complex and difficult case.

Research has consistently revealed that masculine generics evoke a *male bias in mental representations* and make readers or listeners think more of male than female exemplars of a person category (Stahlberg et al., 2007). Effects of linguistic forms on mental representations were measured with the help of various experimental methodologies, for instance, (1) completing sentences with different pronouns and nouns (e.g., *he*, *she*, *he/she*, *the lawyer*, *the client*; Jacobson and Insko, 1985), (2) writing stories about fictitious people following an introductory sentence in the masculine or in gender-fair wording (Heise, 2000), (3) naming female or male representatives (e.g., favorite musician) in response to either masculine nouns or combinations of feminine and masculine forms (Stahlberg et al., 2001), (4) estimating the proportion of women and men in certain roles (e.g., participants at a congress of nutritionists versus geophysicists; Braun et al., 1998), (5) measuring reading time as an indicator of fit between sentences about social groups denoted by nouns with different grammatical gender and sentences that contained a reference to the social group that qualified the group members as female, male, or neither one (Irmen and Roßberg, 2004), or (6) measuring reaction times when classifying gender-related (e.g., *she*, *he*) or neutral pronouns (e.g., *it*, *me*) as female or male after perceiving gender-related (e.g., *mother*, *father*, *nurse*, *doctor*) or gender-neutral primes (e.g., *parent*, *student*; Banaji and Hardin, 1996). The masculine bias in language has been observed in English (e.g., Crawford and English, 1984; Hamilton, 1988; Gastil, 1990; Ng, 1990), French (e.g., Chatard et al., 2005; Gabriel et al., 2008), German (e.g., Heise, 2000; Stahlberg et al., 2001; Braun et al., 2005; Irmen, 2007), Italian (e.g., Cacciari and Padovani, 2007), Polish (e.g., Bojarska, 2011), and Spanish (Carreiras et al., 1996). In a study with German and Belgian school children, the grammatical form of job titles was found to influence the children’s perceptions of typically male jobs: when occupations were presented in the masculine (e.g., German *Ingenieure*, masc.pl ‘engineers’) the mental accessibility of female jobholders was lower than with feminine-masculine word pairs (e.g., *Ingenieurinnen und Ingenieure*, fem.pl and masc.pl ‘[female and male] engineers’; Vervecken et al., 2013). In another study, adult speakers as well envisaged more men in an occupation when job advertisements included more masculine than feminine forms (Gaucher et al., 2011). In all, both the range of methods as well as the number of languages for which the male bias of masculine generics has been documented attests to the validity of the finding.

In general, different strategies can be used to make language gender-fair and avoid detrimental effects of masculine generics: neutralization, feminization and a combination of the two. Which strategy is the appropriate one depends on the type of language concerned (grammatical gender language, natural gender language, or genderless language, Bußmann and Hellinger, 2003).

In the framework of *neutralization* gender-marked terms are replaced by gender-indefinite nouns (English *policeman* by *police officer*). In grammatical gender languages, gender-differentiated forms are replaced, for instance, by epicenes (i.e., forms with invariant grammatical gender which refer to female as well as male persons; e.g., German *Staatsoberhaupt*, neut. ‘head of state’ or *Fachkraft*, fem. ‘expert’ in German). Neutralization has been recommended especially for natural gender languages (e.g., Hellinger and Bußmann, 2003; for English; Norwegian; Danish) and genderless languages (e.g., Engelberg, 2002, for Finnish), as it is fairly easy to avoid gender markings in these languages. Thus, neither generic *he* nor the combination *he/she*, but “singular *they* is the dominant epicene pronoun in modern written British English. However, despite its use, singular *they* has never been endorsed by institutions of the English language, such as major dictionaries and style guides (although many style guides now reject generic *he…*)” (Paterson, 2014, p. 2). Recently, a gender-neutral third person pronoun was invented in Swedish: *hen.* This neologism first appeared in 2012 in a children’s book where it served as an alternative to the gender-marked pronouns ‘she’ *(hon)* and ‘he’ *(han;* Gustafsson Sendén et al., 2015).

In contrast, *feminization* is based on the explicit inclusion of women. Thus, masculine generics are replaced by feminine-masculine word pairs (e.g., German *Elektrikerinnen und Elektriker* ‘[female and male] electricians’; Polish *nauczycielki i nauczyciele* ‘[female and male] teachers’) or abbreviated forms with slashes (e.g., German *Elektriker/in*; Polish *nauczyciel/ka*) or brackets (e.g., *Elektriker[in]*; *nauczyciel[ka]*). Feminization has been recommended for grammatical gender languages such as German, Spanish, Czech, and Italian (Hellinger and Bußmann, 2003; Moser et al., 2011), usually in combination with neutralizing in order to avoid overly complex sentence structures.

However, feminization is not always advantageous for women. The Italian feminine suffix -*essa*, for example, has a slightly derogatory connotation (e.g., Marcato and Thüne, 2002). Accordingly, a woman introduced as *professoressa* ‘female professor’ was perceived as less persuasive than a man or than a woman referred to with the masculine form *professore* (Mucchi-Faina, 2005). Masculine terms used in reference to a female jobholder were associated with higher status than feminine job titles with -*essa* (Merkel et al., 2012). Another example is the German (originally French) suffix-*euse* or -*öse.* Feminine terms such as *Masseuse* ‘(female) masseur’ and *Frisöse* ‘(female) hair dresser’ evoke sexual or frivolous associations, so that the neutral suffix *-in* is usually preferred, as in *Ingenieur-in* ‘female engineer,’ or *Spediteur-in* ‘female forwarding agent.’ Especially in Slavic languages feminine job titles tend to be associated with lesser status, with rural speech, or with the meaning ‘wife of…’ rather than ‘female job holder’ (for Russian: Doleschal and Schmid, 2001; for Serbian: Hentschel, 2003; for Polish: Koniuszaniec and Blaszkowa, 2003). There are also asymmetries in meaning between feminine and masculine forms, as with Polish *sekretarka* ‘female secretary,’ which designates a personal assistant, whereas the masculine *sekretarz* refers also to a high governmental function. In Polish, the feminine suffix -*ka* not only derives feminine occupational terms (such as *nauczyciel-ka* ‘female teacher’ from masculine *nauczyciel* ‘teacher’) but also words for inanimate objects such as *marynar-ka* ‘jacket’ from masculine *marynarz* ‘sailor.’ Problems of this kind can limit the possibilities of feminization in some languages. Where feminization faces such structural problems, its use is less widespread and may have negative effects (Italian: Mucchi-Faina, 2005; Polish: Formanowicz et al., 2013, 2015). But where feminine suffixes are productive feminization can became a linguistic norm and can be evaluated positively (German: Vervecken and Hannover, 2012).

The focus of early research on GFL was mostly on the masculine bias associated with masculine generics. But although these findings suggest that linguistic asymmetries may have farther-reaching consequences, this line of research has made no further progress until recently. The latest findings are more comprehensive and indicate how linguistic asymmetries may facilitate *(unintended) forms of social discrimination* (Mucchi-Faina, 2005; Stahlberg et al., 2007). For example, adult women were reluctant to apply to gender-biased job advertisements (e.g., English job titles ending in -*man*) and were more interested in the same job when the advertisement had an unbiased form (Bem and Bem, 1973). Also, the likelihood of naming women as possible candidates for the office of chancellor in Germany was found to depend on the grammatical gender of the word ‘chancellor’ in the question (Stahlberg and Sczesny, 2001). When the masculine generic (*Kanzler*) was used, fewer respondents suggested female politicians compared to a combination of masculine and feminine form (*Kanzler oder Kanzlerin* ‘[male or female] chancellor’). Moreover, self-evaluation and evaluations by others were found to be influenced by linguistic forms. Thus, girls assumed women to be less successful in typically male occupations when the jobs were described with masculine rather than gender-fair forms, and they were also less interested in these occupations (see also Chatard et al., 2005; Vervecken et al., 2013). Using feminine-masculine word pairs rather than masculine forms for traditionally male occupations boosted children’s self-efficacy (Vervecken and Hannover, 2015). Furthermore, occupations described in pair forms mitigated the difference between ascribed success to female and male jobholders in gendered occupations (Vervecken et al., 2015). Also, women’s perceptions of belonging were found to mediate the effect that women found jobs advertised in the masculine less appealing (Gaucher et al., 2011). Accordingly, women experienced the use of gender-exclusive language during a mock job interview as ostracism (Stout and Dasgupta, 2011). They reported a lower sense of belonging when gender-exclusive language (*he*) was used compared to gender-inclusive (*he or she*) or gender-neutral (*one*) forms. In a study on Austrian German, the wording of job advertisements influenced the evaluation of candidates for leadership positions (Horvath and Sczesny, 2015): men were perceived as fitting a high-status leadership position better than women when a masculine job title was used (*Geschäftsführer*, masc. ‘chief executive officer, CEO’). But when the job ad was gender-fair (*Geschäftsführerin/Geschäftsführer*, fem./masc. ‘[female/male] CEO’), women and men were judged as equally suited. In the context of a lower-status position (project leader) no differences of this kind occurred.

## Language Policies

Many countries have pledged themselves to an equal treatment of women and men (e.g., the member states of the European Union and associated states in the Treaty of Lisbon- European Commission, 2007), and the use of GFL is widely recommended (Schweizerische Bundeskanzlei, 1996, revised in 2009; UNESCO, 1999; National Council of Teachers of English, 2002; European Commission, 2008; American Psychological Association, 2009). But the implementation of GFL has reached different stages in different countries and speech communities.

In the 1970s, guidelines for GFL were introduced in particular professional domains across national and linguistic boundaries, for example, by the American Psychological Association (1975), by the McGraw-Hill Book Company (1974; see also Britton and Lumpkin, 1977; Sunderland, 2011) and the Macmillan Publishing Company (1975). These guidelines demand that authors of (psychological) articles, books, teaching materials, or fiction treat women and men equally, including the language they use (see also Sadker et al., 1991). Publication guidelines of this kind have been effective, because authors need to follow the rules if they want to see their manuscripts published. In texts written by Australian academics (Pauwels, 2003), for example, masculine generic pronouns were infrequent. Similarly, an analysis of American Psychological Association journal articles from the years 1965–2004 revealed a complete absence of generic *he* from 1985 onward, even if the articles still contained other, more subtle gender biases such as androcentric reporting in tables and graphs (Hegarty and Buechel, 2006).

In 1987 representatives of Canada and the Nordic countries argued for an adoption of GFL by the *United Nations Educational, Scientific and Cultural Organization*. This resulted in the creation of guidelines in UNESCO (1999). UNESCO’s position in favor of GFL is described in their gender equality guidelines: “This development indicated a growing awareness that language does not merely reflect the way we think: it also shapes our thinking. If words and expressions that imply that women are inferior to men are constantly used, that assumption of inferiority tends to become part of our mindset; hence the need to adjust our language when our ideas evolve” (UNESCO, 2011, p. 4). The document not only became the most widely recognized international standard for GFL, it also regulates language use in internal documents and publications of UNESCO. Similar guidelines for publications were issued by the European Commission (2008), referring to all working languages of the European Union (EU). Yet, the standards promoted by UNESCO and the EU do not regulate language use in the different countries and are not considered mandatory within their member states.

The availability of GFL policies and the extent of their implementation, that is, their dissemination and execution, also vary considerably between countries (Moser et al., 2011). In Italy, for instance, guidelines for GFL were issued in Sabatini (1987), in the German-speaking area most guidelines appeared in the 1990s (e.g., Hellinger and Bierbach, 1993; Schweizerische Bundeskanzlei, 1996; revised in 2009), and in the Czech Republic guidelines were published only in Valdrová et al. (2010). In other countries such as Poland there are as yet no official guidelines at all. While in some states GFL policies are mentioned only on the website of a ministry (e.g., Czech Republic; Valdrová et al., 2010), use of GFL is mandatory in job ads and public administration in Austria. Since the 1990s the German Duden dictionaries, for example, have included not only the masculine form of personal nouns and job titles but routinely cite the corresponding feminine forms (Kunkel-Razum, 2004). The dictionary lists even feminine forms that are infrequent in texts. An example is the word *Päpstin* ‘female pope,’ which has been listed in the *Grosses Wörterbuch der deutschen Sprache* (Large dictionary of the German language) from the year 1999 onward, even though obviously there never was a female pope in the history of the Catholic Church (Kunkel-Razum, 2004). Moreover, the Duden editors decided to include a chapter on the “equal treatment of women and men in language” in the ninth volume of the series *Richtiges und gutes Deutsch* (Correct and good German). The chapter describes the linguistic potential which the German language offers for speaking or writing in a gender-fair way.

In the German-speaking countries, language policies have become part of the organizational culture of various institutions such as universities and administrations (e.g., Schweizerische Bundeskanzlei, 1996, revised in 2009; Merkel, 2011; Swiss Federal Institute of Technology Zurich, 2011; Gendup – Zentrum für Gender Studies und Frauenförderung, 2012). Even so, Austria is the only country where the use of GFL in job advertisements is strictly prescribed and companies are fined for failing to address both genders in their job ads (Bundesministerium für Frauen und Öffentlichen Dienst, 2009). This may be the reason why the proportion of job ads worded in GFL differs between Austria and German-speaking Switzerland: only 9% of Austrian job advertisements contain masculine generics, whereas it is 27% in Switzerland (Hodel et al., 2013).

School and education are of particular importance for the implementation of GFL. In most countries there are few official GFL guidelines for authors of educational materials (Eurydice, 2009) and regulations concerning schoolbooks exist only in certain countries (e.g., Germany, Ireland, or Iceland). Similarly, only a few countries require schoolbooks to be officially evaluated or approved. In the UK, for example, educational authorities do not monitor teaching materials and schools choose them autonomously. Today German schoolbooks for mathematics and German mostly use gender-neutral forms, followed by masculine generics and feminine-masculine word pairs, (Moser and Hannover, 2014). The two gender-fair options together (word pairs and neutralizing) outweighed the masculine in the schoolbook sample that was analyzed. Since earlier studies on German schoolbooks (e.g., Lindner and Lukesch, 1994; Preinsberger and Weisskircher, 1997) reported a predominance of masculine generics, this finding indicates an increase of GFL in schoolbooks. In some of the texts, however, feminine-masculine word pairs were mixed with masculine generics (see also Markom and Weinhäupl, 2007). This inconsistency is problematic because in the presence of word pairs masculine forms may be understood as referring to male persons only (e.g., Gabriel et al., 2008).

## Individual Language Behavior

Apart from language structures and country-specific aspects, there are a number of factors that make individuals use or reject GFL. One major factor is the novelty of gender-fair forms, which conflicts with speakers’ linguistic habits (Blaubergs, 1980). As long as this is the case, people may experience GFL as irritating, and consequentially may refrain from using it. This could explain why negative effects of GFL have been found especially in the initial phases of language reform such as, for instance, in English in the 1990s (McConnell and Fazio, 1996), and in Italian and Polish in the beginning of the 21st century (Mucchi-Faina, 2005; Merkel et al., 2012; Formanowicz et al., 2013).

Moreover, initiatives for GFL were first instigated by activist movements (e.g., Silveira, 1980; Pusch, 1984) and for that reason often met with negative reactions (Blaubergs, 1980; Parks and Roberton, 1998; Formanowicz et al., 2013). It is conceivable that individual reactions toward GFL are not only caused by its novelty, but also depend on attitudes toward gender arrangements (Jost and Kay, 2005; Carney et al., 2008), for conservative political attitudes are associated both with lesser openness for novelty (Carney et al., 2008) and with stronger support for traditional gender arrangements (Jost et al., 2003, 2008; Hoyt, 2012). Thus, speakers of Polish with more conservative attitudes devaluated female job applicants referring to themselves with a feminine job title compared to female and male applicants using a masculine job title (Formanowicz et al., 2013).

Another factor for individual speakers’ use of GFL might be speakers’ gender: women could be expected to hold more favorable attitudes toward GFL than men and they might be more inclined to use it in their own speech. However, research findings on this point are mixed. While in some studies men rejected GFL more than women did (e.g., Parks and Roberton, 2004; Douglas and Sutton, 2014), other studies found no gender difference in attitudes toward GFL (e.g., Sczesny et al., 2015). Gender differences were mediated by participants’ attitudes toward women, which were, in turn, driven by more comprehensive ideologies that justified the social gender hierarchy (i.e., gender-specific system justification and social dominance orientation; Douglas and Sutton, 2014).

Language use has been viewed as associated with speakers’ *sexist attitudes*, so much so that the use of sexist language has been regarded as an example of subtle sexism (Swim et al., 2004). Modern sexism, for instance, is a view that denies that women are still discriminated against and disapproves of policies promoting gender equality (Swim et al., 1995). In fact, participants with modern sexist beliefs were found to use more traditional, gender-unfair language (Swim et al., 2004). Correspondingly, speakers with stronger sexist attitudes toward women used gender-fair pronouns less frequently than speakers with less sexist attitudes (Jacobson and Insko, 1985). Speakers with progressive gender role perceptions, on the other hand, exhibited a tendency to avoid sexist language when writing an essay (McMinn et al., 1991).

This raises the question how sexist or non-sexist ideologies translate into actual language behavior. Spontaneous use of GFL was found to be guided by explicit intentions to use GFL as well as more implicit processes involving use of GFL in the past (Sczesny et al., 2015). GFL use was not predicted directly by sexist beliefs but by intentions and habits. In other words, sexist speakers do not avoid GFL just because they are reluctant to change their linguistic habits, they deliberately employ a form of language that treats males as the norm and makes women less visible. Habits guide speakers’ linguistic behavior without their being aware of it (Sczesny et al., 2015), and learning processes play a role for GFL to become a habit. *S*peakers who grew up with schoolbooks using predominantly masculine generics (e.g., English: Hellinger, 1980; Campbell and Schram, 1995; Lee and Collins, 2008; German: Lindner and Lukesch, 1994; Preinsberger and Weisskircher, 1997) tend not to question this usage. But once speakers have acquired the habit of using GFL they will rely on this language form. Establishing GFL habits via teaching and practicing current linguistic standards (e.g., Duden; Kunkel-Razum, 2004) is a promising approach which should follow the initial phase of GFL implementation and may reduce political controversies. In this sense, a prevalence of GFL in the media could also promote the use of GFL by individual speakers.

So far, few studies have investigated how speakers can be made to use and approve of GFL. After training interventions, speakers of English used slightly more gender-fair pronouns in completing sentences than non-attendants (McMinn and Foster, 1991; McMinn et al., 1991; Prentice, 1994). Their attitudes, however, did not change (Prentice, 1994). German speakers as well used more GFL after being exposed to arguments for GFL than in a control condition (Koeser and Sczesny, 2014), but this did not affect their attitudes toward GFL. Interestingly, merely reading texts in gender-fair wording can also increase speakers’ own use of GFL: female speakers of German employed more gender-fair forms after reading a gender-fair text than after other texts, but there was no such effect for men (Koeser et al., 2015). Male speakers increased their use of gender-fair forms only when their attention was drawn to GFL forms. These findings indicate that it is more difficult to change attitudes than to promote speakers’ actual use of GFL.

## Overcoming Gender Stereotyping And Discrimination With Gender-Fair Language?

Over the past decades, a large body of research—based on various experimental methodologies, from storytelling to measuring reaction times—has confirmed the influence of linguistic forms on the accessibility of mental representations of women and men (see Stahlberg et al., 2007). Regardless of language structure and of the ease of implementing GFL (Bußmann and Hellinger, 2003), a consistent finding is that speakers do not understand masculine forms as referring to both genders equally but that they interpret them in a male-biased way. This underscores the importance of implementing GFL in everyday language and of using it consistently, so that speakers take up this usage in their own texts and utterances.

How successful have the respective language policies been so far? In *natural gender languages*, neutralization has been fairly easy to adopt and implement (e.g., English, Danish). But even in these language communities people are guided by their knowledge about typical gender distributions in social roles. Thus, English readers tend to associate different occupations or role nouns with men or women, since gender stereotypes are incorporated in their mental representations (Oakhill et al., 2005); and even though there are fewer gender-marked forms in natural gender languages, masculine generics exist and their use can result in social discrimination (Stout and Dasgupta, 2011). In *grammatical gender languages*, feminization as the main strategy of GFL still poses challenges. This is especially true for some languages, e.g., Italian (Merkel et al., 2012) and Slavic languages (Koniuszaniec and Blaszkowa, 2003), where the creation of feminine forms can be problematic, as outlined above. Refusal of GFL can still be observed (Formanowicz and Sczesny, 2014). Such disadvantages are likely to occur while the change is in progress (Formanowicz et al., 2015).

Moreover, our review suggests that—independent of language structure—GFL is more frequent and more accepted when it is backed by official regulations and when the use of biased language is sanctioned in some way (e.g., in official publications or texts; American Psychological Association, 1975, 2009; Bundesministerium für Frauen und Öffentlichen Dienst, 2009; see Hodel et al., 2013). The relationship between policy-making and social change is surely bidirectional. On the one hand, gender equality movements and their demands find their way into legislation. On the other hand, official regulations may stipulate social change by facilitating the internalization of new norms and enforcing their execution. Public discussions over policies also enhance public awareness for GFL (see above the singular pronouns *they* in English and *hen* in Swedish). The contribution of language reforms to gender equality in a society/speech community can best be assessed with investigations that compare countries sharing the same language (e.g., French in Canada and in France) as well as countries with different languages (e.g., Polish and German, two grammatical languages at different stages of implementing GFL). Although there have been some attempts at this type of research (Formanowicz et al., 2015; Gustafsson Sendén et al., 2015) more research is needed to evaluate the effectiveness of language-related policies and provide an evidence-based rationale for policy-making.

As mentioned above, speakers’ use of GFL results from deliberate processes, involving attitudes and intentions, and habitual processes, involving repetition of past behavior (Sczesny et al., 2015). Both types of processes are relevant for the successful implementation of GFL. Despite the various guidelines and legal regulations for GFL that exist on global and national levels, spontaneous use of GFL by individual speakers still seems to be infrequent. For instance, use of GFL in a gap-filling task was quite low among speakers of German from Germany and Switzerland, although GFL policies are fairly advanced in both countries. Most of the participants used more masculine generics than gender-fair forms. As language use is an action performed in a wide range of circumstances, future research should also assess the contiguity between behavior and context. Speakers may employ GFL when writing official texts, for instance, but not when talking or writing to friends. Moreover, attitudes, norms, and intentions concerning GFL in general seem to be only moderately favorable. Even though positive arguments for GFL can help to promote a change in language behavior (Koeser and Sczesny, 2014), future research should attempt to identify factors that are crucial for a deliberate use of GFL. For instance, it might be worthwhile to determine the content and strength of attitudes in different groups of speakers, namely speakers who use GFL regularly compared to speakers who use GFL only occasionally and others who do not use it at all. To gain a more comprehensive understanding of the processes underlying a rejection of GFL, future research could also take a closer look at people’s political attitudes (Formanowicz et al., 2013), their preference for status quo, and their acceptance of traditional gender arrangements (Jost et al., 2008).

In any case, attitudes toward GFL may become more favorable the more frequently and longer GFL has been used (in addition to a mere exposure effect, Zajonc, 1968, see also the existence bias: people treat the existence of something as evidence of its goodness; Eidelman et al., 2009). The role of familiarity for an active use of GFL can best addressed with longitudinal studies. In Sweden, for example, speakers’ attitudes toward the gender-neutral pronoun *hen* have become more positive over time (Gustafsson Sendén et al., 2015). A meta-analytical approach would constitute another way of capturing the dynamics of GFL implementation, taking into account the time when the studies were conducted but also the availability of policies and the structure of the languages concerned. This approach might help to determine whether a language has left the phase where GFL evokes negative associations as well as the role of other factors (such as language policies).

Interventions aiming to increase the use of GFL could focus on a simple repetition of non-sexist expressions, so that these become established habits (Koeser et al., 2015; Wood and Rünger, 2016). This would be a very subtle and implicit way of promoting use of GFL. The development and evaluation of GFL interventions/trainings has not yet been investigated systematically. Future research should take both deliberate and habitual processes of GFL use into consideration, for instance, by analyzing whether children—exposed to and trained in GFL at school (with the help of current schoolbooks)—will later use GFL habitually and consequently hold less gender-stereotypic beliefs.

Finally, there are still obstacles that prevent GFL from becoming a linguistic norm/standard and prevent the change toward an equal treatment of women and men. First, the male bias of linguistic asymmetries in mental representations is backed by a *higher prevalence of men in certain social roles* (e.g., heroes, politicians), which facilitates their cognitive accessibility (Stahlberg and Sczesny, 2001). Once women and men occupy all social roles to a similar extent (see social role theory, which poses that gender stereotype content results from observing women and men in certain societal roles; Eagly, 1987; Bosak et al., 2012), this difference in accessibility should decrease and more gender-balanced mental representations should emerge. Ironically, recent research has documented that linguistic asymmetries prevent girls and women from aspiring to male-dominated roles (see Chatard et al., 2005; Gaucher et al., 2011; Stout and Dasgupta, 2011; Vervecken et al., 2013; Vervecken and Hannover, 2015) and thereby perpetuate the higher accessibility of men in these roles.

Second, the use of gender-unfair language, especially of masculine generics, restricts the *visibility of women* and the *cognitive availability of female exemplars* (Stahlberg et al., 2007), which may be disadvantageous for women (e.g., in personnel selection; Stout and Dasgupta, 2011; Horvath and Sczesny, 2015). However, increasing the visibility of women with the help of novel feminine forms may also have negative consequences and may therefore be avoided, for instance, in women’s professional self-reference (Merkel et al., 2012; Formanowicz et al., 2013). Thus, the avoidance of GFL by women (e.g., avoidance of feminine job titles in grammatical gender languages), in order to protect themselves from ascriptions of incompetence or lower status, also perpetuates the reduction of gender stereotyping and social discrimination.

Third, arguments against GFL have routinely included the presumed difficulty of understanding GFL texts (Parks and Roberton, 1998). Empirical investigations have refuted this argument and have shown that text quality (Rothmund and Christmann, 2002) and cognitive processing were not damaged (Braun et al., 2007). When GFL texts were compared to (generic) masculine texts, there were no differences in readability and esthetic appeal (Blake and Klimmt, 2010). In all, the empirical evidence does not confirm the alleged disadvantage of GFL. Yet, these findings and the scientific evidence for serious disadvantages of masculine generics (see above) have largely been ignored in political controversies and public discussions about GFL. In all, there is a lack of transfer of scientific knowledge which prevents the understanding of linguistic asymmetries as part of a broader gender imbalance and hinders social change. Education and policy-making therefore need to increase the efforts of circulating new scientific insights about GFL to break the vicious circle of ill-informed controversies and discussions about GFL.

At first glance linguistic gender asymmetries seem to affect mostly women. When masculine forms are used it is women who are seen as less prototypical category exemplars, it is women who feel less adequate or are less preferred as job candidates, and it is women who profit from GFL. Therefore, the question arises whether GFL benefits men as well. First, the introduction of GFL might represent a particular challenge for men. In a study by Crawford and English (1984) both male and female participants read a text whose title contained either masculine generics (*Psychologist and his work?*) or GFL (*Psychologist and their work?*) and were to recall the text after 2 days. As the results showed, men’s recall was better in the masculine and women’s recall in the GFL condition. This finding indicates that learning to use GFL involves more than overcoming linguistic novelty. For men, GFL means an unwelcome loss of their privileged position in language. Only in few situations have they something to gain through GFL. If all job advertisements would contain GFL, for instance, men might be more included in traditionally female jobs which used to be referred to in the feminine. Future research should also consider the perspective of men and examine how GFL can turn into a win–win situation for women and men in modern societies.

To conclude, past research has revealed that GFL has the potential to make significant contributions to the reduction of gender stereotyping and discrimination. But as the body of existing evidence is based mainly on experimental paradigms with different kinds of measures, future research should take a closer look on people’s actual language use in everyday life (e.g., in conversations, in the classroom, in social media or organizational communication). Moreover, it will be fruitful to further investigate the dynamics of GFL usage and its effects from cross-linguistic and cross-cultural perspectives (see above the *Marie Curie Initial Training Network - Language, Cognition, and Gender, ITN LCG*, which can be regarded as a first step in this direction). Speakers’ willingness to use GFL in everyday life is crucial in order to profit from the impact of GFL on the (linguistic and social) treatment of women and men in society. But a deliberate effort is required before the use of GFL can become habitual. Education and policy-making can facilitate these processes. When employed consistently over a longer period, and especially when supported by well-informed controversies and discussions, GFL will contribute even more to the reduction of gender stereotyping and discrimination and may thus function as another barometer for change (like the decrease in gender-stereotypical social perception of leadership, Schein, 2001).

## Conflict of Interest Statement

The authors declare that the research was conducted in the absence of any commercial or financial relationships that could be construed as a potential conflict of interest. The reviewer Simona Mancini and handling Editor Manuel Carreiras declared their shared affiliation, and the handling Editor states that the process nevertheless met the standards of a fair and objective review.

## Footnotes

1 In the literature, GFL is also referred to with other terms, e.g., *gender-neutral language*: Sarrasin et al. (2012); *gender-inclusive language*: Stout and Dasgupta (2011); *non-sexist language*: Douglas and Sutton (2014).

2 According to McConnell-Ginet (2013), however, the concept of natural gender language is a myth, and she suggests calling the respective languages “notional” gender languages, since, for example in English, “concepts and ideas about biological sex matter at least as much as sex itself to the choice of English third-person pronouns.” (p. 3).
